# Supplementary material for: Health system cost of breast cancer treatment in Addis Ababa, Ethiopia
Source: PLoS One. 2022 Oct 6;17(10):e0275171. doi: 10.1371/journal.pone.0275171 (PMC9536570; doi:10.1371/journal.pone.0275171)
Supplement: Attachment — Submitted filename: Responses to reviewer 2nd round.docx [file pone.0275171.s006.docx]

**Responses to reviewers**

**Reviewer #2 suggestion:**

Thank you for addressing the previous comments. Kindly consider to convert the point form sentences in the Introduction part into paragraph. Please check any typographical errors in the document (Font color etc).

**My response:** I did accordingly as follow

Accordingly, the basic stages of breast cancer are stage 0, I, II, III and IV and each of them are elaborated as follow**.**

Breast cancer Stage 0 is defined as the cancer cells are not non-invasive. It is also known as ductal carcinoma in situ. This means there is no evidence of cancer cells or non-cancerous abnormal cells breaking out of the area of the breast where they began, or of getting through to or invading neighboring normal tissue.

Breast cancer stage I refers to an extremely early stage of invasive cancer. Tumor cells have spread to normal surrounding breast tissue but are still contained in a small area at this point.

Breast cancer stage II cancer is defined as cancer that has spread beyond a specific region of the breast. It shows the number of lymph nodes that may contain cancer cells.

Breast cancer stage III breast cancer means that the cancer has spread further into the breast or that the tumor is larger than in earlier stages.

The most advanced stage of breast cancer is stage IV. It has spread to nearby lymph nodes as well as distant areas of the body outside of the breast. This means it could affect the organs, such as your lungs, liver, or brain, as well as your bones (1-5).

**References**

1.	IARC, UICC. User’s Guide to ESSENTIAL TNM (E TNM). IARC and UICC; 2022.

2.	ASCO. Breast Cancer: Stages: Cancer.net; 2021 [cited <https://www.cancer.net/cancer-types/breast-cancer/stages> 29/08/2022].

3.	Suzanne Verity. WebMD [Internet]2021.

4.	American Cancer Society. Cancerorg [Internet]2021. [cited 29/08/2022].

5.	UICC. TNM Classification of Malignant Tumors. James D. Brierley (Editor) MKGE, Christian Wittekind (Editor), editor. UK: UICC; 2017.

6.	Cleveland Clinic. Breast Cancer Ohio, USA: Cleveland Clinic; n.d. [cited <https://my.clevelandclinic.org/health/diseases/3986-breast-cancer> 25th July 2022].
